# Outcomes of allogeneic haematopoietic cell transplantation for myelofibrosis in children and adolescents: the retrospective study of the EBMT Paediatric Diseases WP

**DOI:** 10.1038/s41409-024-02286-3

**Published:** 2024-04-16

**Authors:** Jacek Wachowiak, Jacques-Emmanuel Galimard, Arnaud Dalissier, Rawad Rihani, Hawazen AlSaedi, Robert F. Wynn, Jean-Hugues Dalle, Régis Peffault de Latour, Petr Sedlacek, Adriana Balduzzi, Thomas Schroeder, Ivana Bodova, Marta Gonzalez Vicent, Bernd Gruhn, Rose-Marie Hamladji, Gergely Krivan, Katharine Patrick, Agnieszka Sobkowiak-Sobierajska, Polina Stepensky, Ali Unal, Persis Amrolia, Antonio Perez Martinez, Fanny Rialland, Mahmoud Aljurf, Antonella Isgro, Amos Toren, Marc Bierings, Selim Corbacioglu, Krzysztof Kałwak

**Affiliations:** 1 Department of Pediatric Oncology, Hematology and Transplantology, University of Medical Sciences, Poznan, Poland; 2 EBMT Paris Study Office, Paris, France; 3 Department of Haematology, Saint Antoine Hospital, INSERM UMR 938, Sorbonne University, Paris, France; 4 Department of Pediatrics, Pediatric Blood and Marrow Transplantation and Cellular Therapy Program, King Hussein Cancer Center, Amman, Jordan; 5 Department of Pediatric Hematology and Oncology, King Faisal Specialist Hospital and Research Center, Riyadh, Saudi Arabia; 6 Department of Blood and Marrow Transplant, Royal Manchester Children’s Hospital, Manchester, UK; 7 Robert Debré Hospital and Université de Paris, Paris, France; 8 Department of Hematology and Stem Cell Transplantation, Saint Louis Hospital, Paris Cité Université, Paris, France; 9 Department of Pediatric Hematology and Oncology, University Hospital Motol, Prague, Czechia; 10 Hematopoietic Stem Cell Transplant Unit, Fondazione IRCCS San Gerardo dei Tintori, Monza, Italy; 11 Department of Medicine and Surgery, Milano-Bicocca University, Milano, Italy; 12 Department of Bone Marrow Transplantation, University of Essen, Essen, Germany; 13 University Children’s Hospital, Bratislava, Slovakia; 14 Unidad de Trasplante Hematopoyético, Hospital Niño Jesús, Madrid, Spain; 15 Department of Pediatrics, Jena University Hospital, Jena, Germany; 16 Centre Pierre et Marie Curie, Service Hématologie Greffe de Moëlle, Alger, Algeria; 17 Department of Paediatric Haematology and Stem Cell Transplantation, Central Hospital for Southern Pest, National Institute for Hematology and Infectology, Budapest, Hungary; 18 Sheffield Children’s Hospital, Western Bank, Sheffield, United Kingdom; 19 BMT and Cancer Immunotherapy Department, Hadassah Hebrew University Medical Centre, Jerusalem, Israel; 20 Department Hematology and Bone Marrow Transplant Center Erciyes University Medical School, Kayseri, Turkey; 21 Great Ormond Street Hospital for Children NHS Foundation Trust, London, UK; 22 University Hospital La Paz, Madrid, Spain; 23 Hotel Dieu, CHU Nantes Dept. D’Hematologie, Nantes, France; 24 King Faisal Specialist Hospital & Research Centre (Adult), Riyadh, Saudi Arabia; 25 Fondazione IME Policlinico Tor Vergata Rome, Rome, Italy; 26 Edmond & Lily Safra Children’s Hospital, Division of Pediatric Hematology, Oncology & BMT, Sheba Medical Center, Tel_Hashomer, Israel; 27 Princess Maxima Center, University Hospital for Children (WKZ), Stem cell transplantation, Utrecht, The Netherlands; 28 Department of Pediatrics, Division of Pediatric Oncology, Stem Cell Transplant, University Hospital of Regensburg, Regensburg, Germany; 29 Department and Clinic of Pediatric Oncology, Hematology and Bone Marrow Transplantation, Wroclaw Medical University, Wroclaw, Poland

**Keywords:** Diseases, Myeloproliferative disease

## Abstract

This retrospective study evaluated 35 children (median age 5.2 years; range 0.4–18) with myelofibrosis (MF), including 33 with primary myelofibrosis and 2 with secondary myelofibrosis transplanted from matched sibling donor (MSD) (*n* = 17) or non-MSD (*n* = 18) between 2000 and 2022. Conditioning was usually chemotherapy-based (*n* = 33) and myeloablative (*n* = 32). Fifteen patients received bone marrow (BM), 14 haematopoietic cells (HC) from peripheral blood (PB), and 6 from cord blood (CB). Day +100 acute GvHD II–IV incidence was significantly lower after MSD-haematopoietic cell transplantation (MSD-HCT) than after non-MSD-HCT [18.8% (4.3–41.1) vs 58.8% (31–78.6); *p* = 0.01]. Six-year non-relapse mortality (NRM) was 18% (7.1–32.8), relapse incidence was 15.9% (5.6–30.9), progression-free survival (PFS) was 66.1% (47–79.7), GvHD-free relapse-free survival was 50% (30.6–66.7), and overall survival (OS) was 71.1% (51.4–84). Six-year PFS and OS were significantly higher after BM transplantation compared to HCT from other sources [85.1% (52.3–96.1) vs 50.8% (26.3–71), *p* = 0.03, and 90.9% (50.8–98.7) vs 54% (28.1–74.2), *p* = 0.01, respectively], whereas NRM was significantly lower [0% vs 32% (12.3–53.9); *p* = 0.02]. This first multicentre study on outcomes of allogeneic HCT in children with myelofibrosis proves feasibility and curative effect of transplantation in these children, suggests that bone marrow transplantation is associated with better outcomes, and indicates the need for further studies.

## Introduction

In contrast to the adult population, the classical *BCR::ABL1*-negative myeloproliferative neoplasms (*BCR::ABL1*-neg MPNs), i.e. polycythaemia vera (PV), essential thrombocythaemia (ET) and primary myelofibrosis (PMF) [1] are very rare in the paediatric population with an incidence approximately 100×lower than in adults [2].

The discovery of three driver mutated genes *JAK2*, *CALR* and *MPL* was a major landmark in the understanding of *BCR::ABL1*-neg MPNs [3]. These three mutations are detected in more than 80% of adult patients and correlate with clinical characteristics, disease-related complications as well as with prognosis and therefore are helpful in treatment stratification [4, 5]. However, in children the mentioned driver mutations occur in less than 50% of them, posing a significant diagnostic challenge given the lack of an objective clonal marker [6, 7]. In addition, the thrombocytosis and erythrocytosis not related to clonal myeloproliferation are observed much more frequently in the paediatric population than in adults. Apart from this, in children the normal range of haemoglobin level, haematocrit value and erythrocytes number depend on the child’s age. Thus, in children and adolescents the utility of the WHO diagnostic criteria of ET, PV, and PMF is limited [1]. Therefore, in the majority of paediatric patients the diagnosis of *BCR::ABL1*-neg MPN is still difficult [8, 9].

As a consequence of the above mentioned factors, the opportunities to perform prospective clinical trials concerning *BCR::ABL1*-neg MPNs are severely limited in the paediatric population, and in contrast to adults, there is still a lack of established prognostic criteria and treatment recommendations based on these criteria, including specific recommendations for allogeneic haematopoietic cell transplantation (allo-HCT) in paediatric patients suffering from *BCR::ABL1*-neg MPNs [7, 9].

In adults PMF and post-ET/PV myelofibrosis (post-ET/PV MF) are *BCR::ABL1*-neg MPNs with the worst survival rates, but allo-HCT can cure a substantial number of patients, especially those younger than 70 years and with a median survival expectation of less than 5 years [10].

In children and adolescents myelofibrosis (MF) is the rarest type of *BCR::ABL1*-neg MPNs [2, 6, 7], and so far there was no study analysing the outcomes of allo-HCT in a larger group of paediatric patients with MF, and therefore the data on allo-HCT outcomes in them remain casuistic and scant [11–14].

For this reason this retrospective, multicentre study on transplant-specific characteristics and outcomes of allo-HCT performed in paediatric patients with PMF or post-ET/PV MF between 2000 and 2022 and reported to the European Society for Blood and Marrow Transplantation (EBMT) Registry was carried out within the EBMT Paediatric Diseases Working Party.

## Methods

This was a retrospective EBMT Registry-based analysis approved by the EBMT Pediatric Diseases Working Party and performed in a cohort of children and adolescents ( < 18 years) receiving the first allogeneic haematopoietic cell transplantation (allo-HCT) for primary myelofibrosis (PMF), post-essential thrombocythaemia or post-polycythaemia vera secondary myelofibrosis (post-ET/PV MF), and transplanted between January 1^st^ 2000 and December 31^st^ 2022.

The following outcome measures were analysed in the studied cohort of paediatric patients: cumulative incidence of neutrophil recovery defined as an absolute granulocyte count (ANC) greater than 0.5 × 10^9^/l for three consecutive days unsupported by granulocyte colony stimulating factor; platelets recovery defined as a platelet count above 20 ×10^9^/l for three consecutive days (with no platelet transfusions seven days prior) [15, 16]; acute graft-versus-host disease (aGvHD) and chronic GvHD (cGvHD) occurrence graded according to standard criteria [17, 18]; non-relapse mortality (NRM) defined as death without evidence of relapse or progression; relapse incidence (RI) defined as the time from transplantation to first occurrence of relapse or progression; progression-free survival (PFS) defined as the time from transplantation to relapse or death due to neoplasm; GvHD-free relapse-free survival (GRFS) defined as the time from transplantation to first event of aGvHD grade III-IV, extensive cGvHD, relapse or death, and overall survival (OS) defined as the time from transplantation to death from any cause.

### Statistical methods

Quantitative variables were described as median, quartile 1 and 3, minimum and maximum. Differences between two groups and quantitative variables were tested using the Wilcoxon test. Qualitative variables were described as number and percentage. Differences between groups and qualitative variables were tested using the Chi-squared test or the Fisher Exact test when Chi-squared test validation was not respected.

OS, PFS, and GRFS were estimated using the Kaplan-Meier method. All outcomes with competing events were estimated using the cumulative incidence function. NRM and RI were mutually competing events. Death and relapse were competing events for GvHD outcomes. Death was a competing event for ANC and platelet recovery, and secondary HCT. Second HCT was a competing event for ANC and platelet recovery. All outcomes were censored at last follow-up. The median follow-up was estimated using the reverse Kaplan-Meier method. Univariable tests of the covariate impact on outcomes were performed using the log-rank test for OS, PFS and GRFS, and Grey’s test for cumulative incidence outcomes. All tests were significant at the level of 0.05 and two-sided. Analyses were performed using the R software version 4.0.2.

### Ethical statement

The study was conducted in accordance with the EBMT Guidelines for Retrospective Studies and the principles of the Declaration of Helsinki. EBMT Centres commit to obtain informed consent with the local regulations applicable at the time of transplantation in order to report pseudoanonymised data to the EBMT.

## Results

### Patient descriptive analysis

A total of 35 children and adolescents who underwent an allo-HCT for myelofibrosis (MF) between 2000 and 2022 were analysed based on data reported to the EBMT Registry (Table 1). PMF was diagnosed in 33 (94.3%) patients and post-ET/PV MF in two (5.7%). There were 12 (34.3%) female patients and 23 (65.7%) male patients. Median age at diagnosis was 3.4 years (range: 0.1–17.7), while at allo-HCT 5.2 years (range 0.4–18). Median time from diagnosis to transplantation was 7.1 months (range: 3.8–136.4). At transplantation the Lansky score was found to be below 90 in 8 of 32 (25%) patients. Before the start of the conditioning regimen the median haemoglobin level was 9.0 g/dl (range: 5.9–11.8), leucocytes count 6.6 × 10^9^/l (range: 1.1–25.6), platelets count 83 × 10^9^/l (range: 1–1505), and 11 (47.8%) patients had palpable splenomegaly. Splenectomy prior to the start of the preparative regimen was performed in only one (3.2%) patient.

**Table 1:** Patient characteristics—total and according to donor type (MSD vs non-MSD) and across analysed era (2000–2007 vs 2008–2022).

Variable	Modality	*N*	MSD	Non-MSD	Test p-value	[2000–2007]	[2008–2022]	Test *p*-value
Total		35	17 (48.6)	18 (51.4)		16 (45.7)	19 (54.3)	
Patient sex	Female	12 (34.3)	5 (29.4)	7 (38.9)	0.55	5 (31.2)	7 (36.8)	0.73
	Male	23 (65.7)	12 (70.6)	11 (61.1)		11 (68.2)	12 (63.2)	
Age at diagnosis	median [IQR]	3.4 [1–11.3]	4.3 [1.5–12.3]	3.2 [0.8–8.4]	0.59	3.1 [0.8–11.1]	3.4 [1.3–9.3]	0.71
	(range)	(0.1–17.7)	(0.1–17.7)	(0.3–16.9)		(0.1–17.4)	(0.4–17.7)	
Age at HCT	median [IQR]	5.2 [2.2–12.8]	4.7 [1.7–13.2]	5.4 [2.8–11.6]	0.72	6 [2.3–12.6]	5.2 [2.3–10.7]	1
	(range)	(0.4–18)	(0.4–18)	(0.9–17.7)		(0.4–17.6)	(0.9–18)	
MF status at HCT	PMF	33 (94.3)	17 (100)	16 (88.9)	Not done	16 (100)	17 (89.5)	1 f
	sMF (post ET)	1 (2.9)	0 (0)	1 (5.6)		0 (0)	1 (5.3)	
	sMF (post PV)	1 (2.9)	0 (0)	1 (5.6)		0 (0)	1 (5.3)	
Months between diagnosis and HCT	median [IQR]	7.1 [3.8–14.4]	4.6 [3.2–7.1]	10.3 [6.3–24.5)	0.004	6.3 [3.3–20.8]	7.1 [4.4–11.4]	0.92
	(range)	(1.2–136.4)	(1.2–30.6)	(3.1–136.4)		(1.2–136.4)	(2–62.4)	
Haemoglobin prior start of conditioning (g/dl)	median [IQR]	9.0 [8.4–10.6]	8.8 [8.4–10.1]	9.2 [8.4–11]	0.41	8.8 [8.2–9.9]	10 [8.7–10.8]	0.29
	(range)	(5.9–118)	(5.9–14.5)	(6.5–118)		(5.9–14.5)	(6–118)	
	missing	10	4	6		2	8	
White blood cells prior start of conditioning (10^9/l)	median [IQR]	6.6 [3.3–10.7]	10.2 [3.6–12.4]	5.9 [3.3–6.7]	0.21	6.8 [2.5–10.6]	5.7 [4.2–10.8]	0.73
	(range)	(1.1–25.6)	(1.1–25.6)	(2.4–10.7)		(1.1–14.5)	(2.9–25.6)	
	missing	10	4	6		2	8	
Platelets prior start of conditioning (10^9/l)	median [IQR]	83 [39–255]	65 [41–412]	103 [30–151.8]	0.43	57 [38.2–126.8]	145 [64.5–373]	0.16
	(range)	(1–1505)	(22–1505)	(1–1191)		(1–510)	(18–1505)	
	missing	10	4	6		2	8	
Palpable splenomegaly prior the start of conditioning	absent	12 (52.2)	7 (53.8)	5 (50)	1 f	8 (57.1)	4 (44.4)	0.68 f
	present	11 (47.8)	6 (46.2)	5 (50)		6 (42.9)	5 (55.6)	
	missing	12	4	8		2	10	
Splenectomy prior start of conditioning regimen	No	30 (96.8)	15 (100)	15 (93.8)	1 f	14 (93.3)	16 (100)	0.48 f
	Yes	1 (3.2)	0 (0)	1 (6.2)		1 (6.7)	0 (0)	
	missing	4	2	2		1	3	
Lansky score	< 90	8 (25)	4 (25)	4 (25)	1 f	4 (28.6)	4 (22.2)	0.70 f
	≥ 90	24 (75)	12 (75)	12 (75)		10 (71.4)	14 (77.8)	
	missing	3	1	2		2	1	

f: Fisher exact test.

*HCT* haematopoietic cell transplantation, *MSD* matched sibling donor, *non-MSD* non-matched sibling donor, *MF* myelofibrosis, *PMF* primary myelofibrosis, *ET* essential thrombocythaemia, *PV* polycythaemia vera, *sMF* secondary myelofibrosis.

Seventeen (48.6%) children were transplanted from matched sibling donors (MSD), all of them (100%) for PMF, and the remaining 18 (51.4%) underwent transplantation from non-MSD, including 16 (88.9%) for PMF and two (11.1%) for post-ET/PV MF (Table 1). The median age of the children transplanted from MSD was 4.3 years (range: 0.1–17.7) at diagnosis and 4.7 years (range: 0.4–18) at transplantation. Among the children transplanted from non-MSD the median age at diagnosis was 3.2 years (range: 0.3–16.9), while the median age at transplantation was 5.4 years (range: 0.9–17.7). The Lansky score was found to be below 90 in 4 of 16 (25%) recipients transplanted from MSD, and in 4 of 16 (25%) transplanted from non-MSD.

Sixteen (45.7%) patients were transplanted between 2000 and 2007, and the remaining 19 (54.3%) between 2008 and 2022 (Table 1). All 16 (100%) patients transplanted between 2000 and 2008 underwent HCT for PMF, while out of 19 patients who received HCT between 2008 and 2022 seventeen (89.5%) underwent transplantation for PMF and 2 (10.5%) for post-ET/PV MF. The median age at diagnosis and at transplantation in children transplanted between 2000 and 2007 was 3.1 years (range: 0.1–17.4) and 6.0 years (range: 0.4–17.6), respectively, and in those who underwent transplantation between 2008–2022 it was 3.4 years (0.4–17.7) and 5.2 years (range: 0.9–18), respectively. Pre-transplant Lansky performance status below 90 was found in 4 of 14 (28.6%) patients receiving HCT between 2000 and 2007, and in 4 of 18 (22.2%) patients transplanted between 2008 and 2022 (Table 1).

### Transplant descriptive analysis

#### Donor and source of haematopoietic cells (HCs)

Seventeen (48.6%) children were transplanted from MSD, and 18 (51.4%) from non-MSD including three (16.7%) from another matched relative donor, three (16.7%) from a haploidentical donor (including one haploidentical cord blood), and 12 (66.7%) from an unrelated donor (UD) including four cord blood and one double cord blood (Table 2). Eight (22.9%) male recipients received hematopoietic cells (HCs) from female donor, including three (17.6%) female MSD and five (27.8%) female non-MSD.

**Table 2:** Transplant characteristics – total and according to donor type (MSD vs non-MSD) and across analysed era (2000–2007 vs 2008–2022).

Variable	Modality	*N*	MSD	Non-MSD	Test p-value	[2000–2007]	[2008–2022]	Test *p*-value
Total		35 (%)	17 (48.6%)	18 (51.4%)		16 (45.7%)	19 (54.3%)	
Donor type	Identical sibling	17 (48.6)	17 (100)		Not done	8 (50)	9 (47.4)	Not done
	Matched other relative	3 (8.6)		3 (16.7)		2 (12.5)	1 (5.3)	
	UD 10/10	3 (8.6)		3 (16.7)		2 (12.5)	1 (5.3)	
	UD 9/10	2 (5.7)		2 (11.1)		0 (0)	2 (10.5)	
	UD (HLA not reported)	2 (5.7)		2 (11.1)		2 (12.5)	0 (0)	
	UCB 5/6	4 (11.4)		4 (22.2)		1 (6.2)	3 (15.8)	
	Double UCB (2/6; 3/6)	1 (2.9)		1 (5.6)		1 (6.2)	0 (0)	
	CB haploidentical	1 (2.9)		1 (5.6)		0 (0)	1 (5.3)	
	Haploidentical	2 (5.7)		2 (11.1)		0 (0)	2 (10.5)	
Female donor to male recipient	No	27 (77.1)	14 (82.4)	13 (72.7)	0.69 f	12 (75)	15 (78.9)	1 f
	Yes	8 (22.9)	3 (17.6)	5 (27.8)		4 (25)	4 (21.1)	
Source of cells	BM	15 (42.9)	11 (64.7)	4 (22.2)	0.006 f	5 (31.2)	10 (52.6)	0.22 f
	PB	14 (40.0)	6 (35.3)	8 (44.4)		9 (56.2)	5 (26.3)	
	CB & double CB	6 (17.1)	0 (0)	6 (33.3)		2 (12.5)	4 (21.1)	
Conditioning regimen	Bu+Cy	16 (45.7)	11 (64.7)	5 (27.8)	Not done	10 (62.5)	6 (31.6)	Not done
	Bu+Cy+Flu	1 (2.9)	0 (0)	1 (5.6)		1 (6.2)	0 (0)	
	Bu+Cy+Flu+Arac	1 (2.9)	1 (5.9)	0 (0)		0 (0)	1 (5.3)	
	Bu+Cy+Mel	3 (8.6)	1 (5.9)	2 (11.1)		2 (12.5)	1 (5.3)	
	Bu+Flu	2 (5.7)	1 (5.9)	1 (5.6)		0 (0)	2 (10.5)	
	Bu+Flu+Thio	1 (2.9)	1 (5.9)	0 (0)		0 (0)	1 (5.3)	
	TBI+Bu+Cy+Flu	1 (2.9)	0 (0)	1 (5.6)		0 (0)	1 (5.3)	
	TBI alone	1 (2.9)	0 (0)	1 (5.6)		1 (6.2)	0 (0)	
	Treo+Flu+Thio	4 (11.4)	0 (0)	4 (22.2)		0 (0)	4 (21.1)	
	Treo+Flu	1 (2.9)	0 (0)	1 (5.6)		0 (0)	1 (5.3)	
	Treo+Cy+Mel	1 (2.9)	0 (0)	1 (5.6)		0 (0)	1 (5.3)	
	Cy+Flu	1 (2.9)	1 (5.9)	0 (0)		0 (0)	1 (5.3)	
	Flu+Mel	2 (5.7)	1 (5.9)	1 (5.6)		2 (12.5)	0 (0)	
Myeloablative regimen	No (CyFlu and FluMel)	3 (8.8)	2 (11.8)	1 (5.9)	1 f	2 (13.3)	1 (5.3)	0.57 f
	Yes	31 (91.2)	15 (88.2)	16 (94.1)		13 (86.7)	18 (94.7)	
	missing	1	0	1		1	0	
GVHD prophylaxis	CSA + MTX	19 (57.6)	13 (81.2)	6 (35.3)	Not done	11 (78.6)	8 (42.1)	Not done
	CSA	6 (18.2)	0 (0)	6 (35.3)		2 (14.3)	4 (21.1)	
	CSA + MMF	3 (9.1)	1 (6.2)	2 (11.8)		0 (0)	3 (15.8)	
	CSA + MMF + TACRO	1 (3)	0 (0)	1 (5.9)		0 (0)	1 (5.3)	
	PT-CY + CSA	1 (3)	0 (0)	1 (5.9)		0 (0)	1 (5.3)	
	ATG only	2 (6.1)	1 (6.2)	1 (5.9)		1 (7.1)	1 (5.3)	
	MTX	1 (3)	1 (6.2)	0 (0)		0 (0)	1 (5.3)	
	missing	2	1	1		2	0	
TCD in vivo	No	9 (27.3)	7 (43.8)	2 (11.8)	0,06 f	5 (35.7)	4 (21.1)	0,44 f
	Yes	24 (71.7)	9 (56.2)	15 (88.2)		9 (64.3)	15 (78.9)	
	missing	2	1	1		2	0	

*MSD* matched sibling donor, *non-MSD* non-matched sibling donor, *UD* unrelated dor, *CB* cord blood, *UCB* unrelated cord blood, *BM* bone marrow, *PB* peripheral blood, *Bu* busulfan, *Cy* cyclophosphamide, *Flu* fludarabine, *Thio* thiothepa, *Mel* melphalan, *Treo* treosulfan, *TBI* total body irradiation, *ATG* anti-thymocyte globuline, *CSA* cyclosporine A, *MFF* mycophenolate mofetil, *TACRO*, tacrolimus, *MTX* methotrexate, *PT* post-transplantation, *TCD* T-cell depletion.

Eleven (64.7%) patients transplanted from MSD and four (22.2%) transplanted from non-MSD (*p* = 0.006) received bone marrow (BM), while peripheral blood (PB) was the source of HCs in six (35.3%) recipients undergoing MSD-HCT and in eight (44.4%) recipients undergoing transplantation from non-MSD (Table 2). A further six (33.3%) patients transplanted from non-MSD obtained HCs from cord blood (CB).

### Conditioning regimen

Thirty-one (91.2%) patients received myeloablative conditioning (MAC), and only three (8.8%) obtained non-myeloablative, reduced intensity conditioning (RIC) (two transplanted from MSD, and one from non-MSD) (Table 2). The conditioning regimen was chemotherapy-based in 33 (94.3%) patients, while FTBI-based was used only in two (5.7%) transplanted from non-MSD, including one patient obtaining FTBI along with busulfan, cyclophosphamide and fludarabine and one obtaining FTBI alone. A busulfan-based regimen was utilized in 24 (68.6%) patients, including 15 (62.5%) receiving HCs from MSD and 9 (37.5%) transplanted from non-MSD. A treosulfan-based regimen was given exclusively to six (17.1%) recipients who underwent non-MSD HSCT.

Among the 16 patients transplanted between 2000 and 2007 a busulfan-based regimen was administered in 13 (81.2%) patients, fludarabine plus melphalan (FluMel) in two (12.5%) and total body irradiation (TBI) in one (6.3%), while in the 19 patients transplanted between 2008 and 2022 busulfan-based regimen was given in 11 (57.9%), treosulfan-based in 6 (31.5%), cyclophosphamide plus fludarabine (CyFlu) in one (5.3%), and TBI+BuCyFlu in one (5.3%) (Table 2).

### GvHD prophylaxis and grading

Data on GvHD prophylaxis were available in 33 children out of 35 (Table 2). In 30 (91.8%) patients GvHD prevention was based on cyclosporin A (CsA). CsA along with short-course methotrexate (MTX) was administered in 19 (57.6%) recipients, including 13 (81.2%) who underwent MSD-HCT and in six (35.3%) who underwent non-MSD-HCT. CsA alone was given in six (18.2%) patients, all of which were transplanted from non-MSD (35.3%). In four (12.1%) recipients CsA was combined with mycophenolate mofetil (MMF), including one (6.2%) who underwent MSD-HCT and in three (16.8%) who underwent non-MSD HSCT. In vivo T-cell depletion (TCD) was performed in 24 (71.7%) patients, among them in 9 (56.2%) before transplantation from MSD and in 15 (88.2%) before transplantation from non-MSD. Complete and detailed data on GvHD prophylaxis for the whole study cohort and according to donor type are presented in Table 2.

### Transplant specific outcomes

#### Neutrophil and platelet engraftment

The day +30 and day +60 cumulative incidence (CI) of neutrophil recovery were 77.1% (58.6–88.1) and 85.7% (67.1–94.2), respectively (Table 3). The day +60 incidence of platelet recovery was 78.1% (58.4–89.3), and after 180 days it was 84.4% (64.7–93.6).

**Table 3:** Neutrophil and platelet engraftment.

Outcomes	N	Number of events	Estimation (95%CI)
ANC ≥ 0.5 × 10^9/L (day +30)	35	27	77.1 (58.6–88.1)
ANC ≥ 0.5 × 10^9/L (day +60)	35	30	85.7 (67.1–94.2)
Platelets ≥ 20 × 10^9/L (day +60)	32	25	78.1 (58.4–89.3)
Platelets ≥ 20 × 10^9/L (day +180)	32	27	84.4 (64.7–93.6)

*ANC* absolute neutrophil count.

### Acute and chronic GvHD

The day +100 CI of aGvHD II–IV was 39.4% (22.7–55.7), and was lower after undergoing MSD-HSCT (18.8%; 4.3–41.1) compared to non-MSD-HSCT (58.8%; 31–78.6) (*p* = 0.01). The day +100 CI of aGvHD III–IV was 9.1% (2.3–21.9) and was lower (0%) after undergoing MSD-HSCT than observed after undergoing non-MSD transplantation (17.6%; 4.1–39), however, the difference was not significant (*p* = 0.08) (Table 4).

**Table 4:** Univariate analysis of acute and chronic GvHD occurence.

Variable	Modality	Total	Day + 100 aGvHD grade II–IV	Day + 100 aGvHD grade III–IV	6 y cGvHD	6 y ext cGvHD
Year of HCT	[2000–2007]	16 (45.7)	50 [23.4–71.8]	12.5 [1.9–33.6]	12.5 [1.7–34.4]	8.3 [0.4–33.3]
	[2008–2022]	19 (54.3)	29.4 [10.1–52]	5.9 [0.3–24.3]	20 [4.3–43.9]	20 [4.3–43.9]
	P value		0.25	0.49	0.46	0.21
Age at HCT (years)	(0–6)	19 (54.3)	41.2 [17.7–63.4]	0	14.7 [2.1–38.7]	10.5 [0.4–39.3]
	[6–18)	16 (45.7)	37.5 [14.6–60.7]	18.8 [4.3–41.1]	18.8 [4.2–41.3]	18.8 [4.2–41.3]
	P value		0.84	0.07	0.61	0.3
Recipient sex	Male	23 (65.7)	38.1 [17.7–58.4]	9.5 [1.5–26.7]	16.9 [3.7–38.4]	11.9 [1.6–33]
	Female	12 (34.3)	41.7 [14–67.7]	8.3 [0.4–32.3]	16.7 [2.3–42.6]	16.7 [2.3–42.6]
	P value		0.9	0.88	0.92	0.53
Donor type	MSD	17 (48.6)	18.8 [4.3–41.1]	0	13.4 [2–35.8]	6.7 [0.4–27.1]
	non-MSD	18 (51.4)	58.8 [31–78.6]	17.6 [4.1–39]	20.4 [4.3–44.8]	21.9 [4.4–47.8]
	P value		**0.01**	0.08	0.65	0.35
Source of HCs	BM	15 (42.9)	42.9 [16.6–67]	7.1 [0.4–28.5]	22.1 [4.8–47.1]	14.3 [2.1–37.5]
	other	20 (57.1)	36.8 [15.9–58.2]	10.5 [1.7–29.1]	12.3 [1.8–33.7]	13.3 [1.8–36.3]
	P value	6 (17.1)	0.88	0.72	0.39	0.66

*HCT* haematopoietic cell transplantation, *aGvHD* acute graft versus host disease, *cGvHD* chronic graft versus host disease, matched sibling donor, *non-MSD* non-matched sibling donor, *BM* bone marrow, *HCs* haematopoietic cells.

The 6-year CI of cGvHD was 16.7% (5.9–32.4), including extensive cGvHD 14.6% (4.2–31.2), which at six years from transplantation did not significantly differ in the children transplanted from MSD (6.7%; 0.4–27.1) and from non-MSD (21.9%; 4.4–47.8) (*p* = 0.35) (Table 4).

Regarding the haematopoietic cells source, i.e. bone marrow vs other sources, there was no significant difference in terms of the day +100 incidence of aGvHD II-IV (42.9%; 16.6–67 vs 36.8%; 15.9–58.2) (*p* = 0.88) or aGvHD III–IV (7.1%; 0.4–28.5 vs 10.5%; 1.7–29.1) (*p* = 0.72). The 6-year incidence of cGvHD (22.1%; 4.8–47.1 vs 12.3%; 1.8–33.7) (*p* = 0.39) and extensive cGvHD (14.3%; 2.1–37.5 vs 13.3%; 1.8–36.3) (*p* = 0.66) were not significantly different (Table 4).

### Non-relapse mortality (NRM)

Six patients died without evidence of relapse, including three patients due to graft failure (13, 32, and 644 days after HCT) and three due to acute GvHD (62, 102, and 109 days after HCT) (Table 5).

**Table 5:** Non-relapse mortality—patient and transplant characteristics, and causes.

Age at HCT	Year of HCT	MF status at HCT	Donor type	Source of HCs	Conditioning regimen	Ex vivo TCD	In vivo TCD	GVHD prophylaxis	Survival (days after HCT)	Cause of death
17.4	2011	PMF	MSD	PB	Cy+Flu	No	ATG	CSA + MTX	13	Graft failure
1.7	2013	PMF	UD 5/6	CB	Treo+Flu+Thio	No	ATG	CSA + MMF	32	Graft failure
4.7	2003	PMF	MSD	PB	Bu+Cy	No	NA	missing	644	Graft failure
11.7	2000	PMF	UD^a^	PB	TBI alone	Yes	NA	missing	62	acute GvHD
17.6	2005	PMF	UD 10/10	PB	Bu+Cy+Flu	Yes	No	CSA + MTX	102	acute GvHD
14.9	2007	PMF	UD (2/6; 3/6)	CB-double	Bu+Cy	No	No	CSA	109	acute GvHD

^a^HLA not reported.

*HCT* haematopoietic cell transplantation, *MF* myelofibrosis, *PMF* primary myelofibrosis, *HCs* haematopoetic cells, *MSD* matched sibling donor, *UD* unrelated donor, *PB* peripheral blood, *CB* cord blood, *Cy* cyclophosphamide, *Flu* fludarabine, *Treo* treosulfan, *Thio* thiothepa, *Bu* busulfan, *TBI* total body irradiation, *TCD* T-cell depletion, *GvHD* graft versus host disease, *ATG* anti-thymocyte globuline, *CSA* cyclosporine A, *MTX* methotrexate, *MFF* mycophenolate mofetil.

**Fig. 1:**
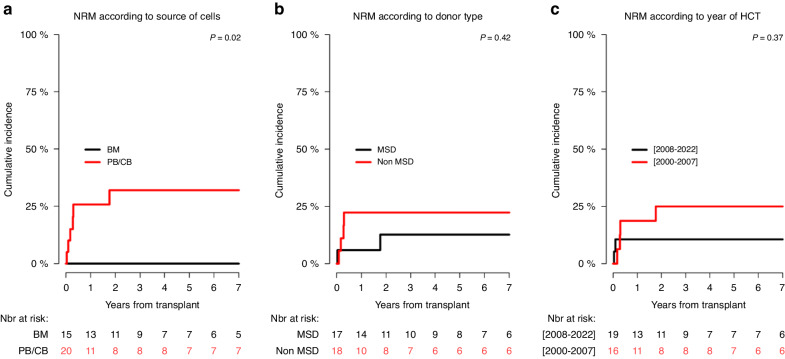
Non-relapse mortality (NRM) curves. Non-relapse mortality cumulative incidence according to: the source of haematopoietic cells (**a**), donor of haematopoietic stem cells (**b**), and transplant period (**c**).

In the whole study cohort the 6-year CI of NRM was 17% (7.1–32.8), while in the children transplanted from MSD it was 12.6% (1.9–34.1), which did not differ significantly from those transplanted from non-MSD (22.2%; 6.6–43.6) (*p* = 0.42), but it was significantly lower in the children obtaining bone marrow (0%) than in those transplanted with hematopoietic stem cells from other sources (32%; 12.3–53.9) (*p* = 0.02) (Table 6, Fig. 1). The 6-year NRM between 2000 and 2007 (25%; 7.2–48.1) was not significantly different from observed between 2008 and 2022 (10.5%; 1.7–29) (*p* = 0.37) (Table 6, Fig. 1).

**Table 6:** Non-relapse mortality cumulative incidence, relapse incidence, progression-free survival (GvHD-free relapse-free survival and overall survival.

Variables	Modalities	n	6y NRM	6y RI	6y PFS	6y GRFS	6y OS
**Total**	[2000–2022]	35	18 [7.1–32.8]	15.9 [5.6–30.9]	66.1 [47–79.7]	50 [30.6–66.7]	71.1 [51.4–84]
**Year of HCT**	[2000–2007]	16 (45.7)	25 [7.2–48.1]	25 [7.2–48.2]	50 [24.5–71]	41.7 [17.4–64.5]	56.2 [29.5–76.2]
	[2008–2022]	19 (54.3)	10.5 [1.7–29]	5.3 [0.3–22.1]	84.2 [58.7–94.6]	62.3 [34–81.3]	89.5 [64.1–97.3]
	P value		0.37	0.18	0.09	0.58	0.09
**Age at HCT**	(0–6)	19 (54.3)	12.8 [1.8–34.8]	12.1 [1.8–33.2]	75.1 [45.6–90.1]	63 [30.9–83.4]	79.5 [48.1–93.1]
	[7–18)	16 (45.7)	25 [7.3–48]	18.8 [4.2–41.3]	56.2 [29.5–76.2]	37.5 [15.4–59.8]	61.1 [32.7–80.5]
	P value		0.3	0.66	0.21	0.053	0.22
**Recipient sex**	Male	23 (65.7)	25 [7.2–48.1]	20.6 [5.9–41.4]	55.6 [31.1–74.4]	40.9 [18.5–62.3]	63.9 [37.9–81.3]
	Female	12 (34.3)	10.5 [1.7–29]	8.3 [0.4–32.4]	83.3 [48.2–95.6]	66.7 [33.7–86]	83.3 [48.2–95.6]
	P value		0.37	0.35	0.12	0.24	0.28
**Months between diagnosis and HCT**	[1–8)	19 (54.3)	16.8 [3.9–37.6]	17.5 [4–38.9]	65.7 [38.8–83]	52.7 [26.7–73.3]	71.3 [44.1–87]
	[9–136]	16 (45.7)	18.8 [4.3–41.1]	13.8 [2–36.7]	67.5 [38.4–85.1]	48.2 [20.8–71.2]	72.7 [42–88.9]
	P value		0.78	0.84	0.87	0.49	0.86
**Donor type**	MSD	17 (48.6)	12.6 [1.9–34.1]	13 [1.9–34.9]	74.3 [45–89.6]	67 [37.9–84.7]	73.7 [43.9–89.3]
	Other	18 (51.4)	22.2 [6.6–43.6]	18.9 [4.1–41.9]	58.8 [32–78.1]	34.9 [12.4–58.8]	69.3 [40.6–86.2]
	P value		0.42	0.56	0.24	0.07	0.6
**Source of HCs**	BM	15 (42.9)	0%	14.9 [2.2–38.9]	85.1 [52.3–96.1]	70.1 [38.5–87.6]	90.9 [50.8–98.7]
	Other	20 (57.1)	32 [12.3–53.9]	17.1 [3.8–38.5]	50.8 [26.3–71]	36.1 [14.5–58.4]	54 [28.1–74.2]
	P value		**0.02**	0.81	**0.03**	0.09	**0.01**

*HCT* haematopoietic cell transplantation, *MSD* matched sibling donor, *HCs* haematopoietic cells, *BM* bone marrow, *NRM* non-relapse mortality, *RI* relapse incidence, *PFS* progression-free survival, *GRFS* GvHD-free relapse-free survival, *OS* overall survival.

### Relapse incidence (RI), progression-free survival (PFS), GvHD-free relapse-free survival (GRFS), and overall survival (OS)

**Fig. 2:**
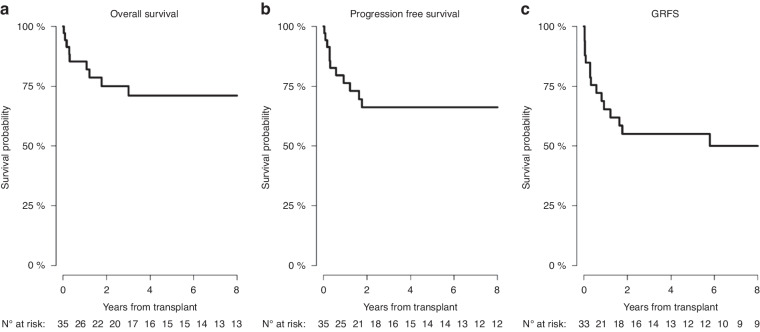
Survival curves for the whole studied group. Overall survival (**a**), progression-free survival (**b**), and GvHD-free relapse-free survival (GRFS) (**c**).

For the whole study group the 6-year RI was 15.9% (7.75.6–30.9), the 6-year PFS was 66.1% (47–79.7), the 6-year GRFS was 50% (30.6–66.7), and the OS was 71.1% (51.4–84) (Table 6, Fig. 2) with a median follow-up of 9.1 years (3.1–11.2).

**Fig. 3:**
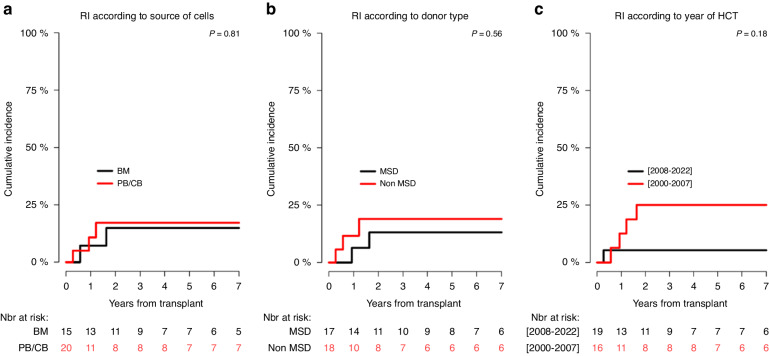
Relapse incidence (RI) curves. RI according to: the source of haematopoietic cells (**a**), donor of haematopoietic cells (**b**), and transplant period (**c**).

**Fig. 4:**
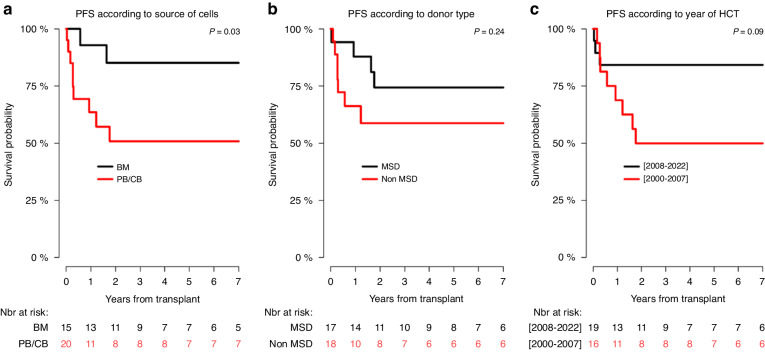
Progression-free survival (PFS) curves. PFS according to: the source of haematopoietic cells (**a**), donor of haematopoietic cells (**b**), and transplant period (**c**).

**Fig. 5:**
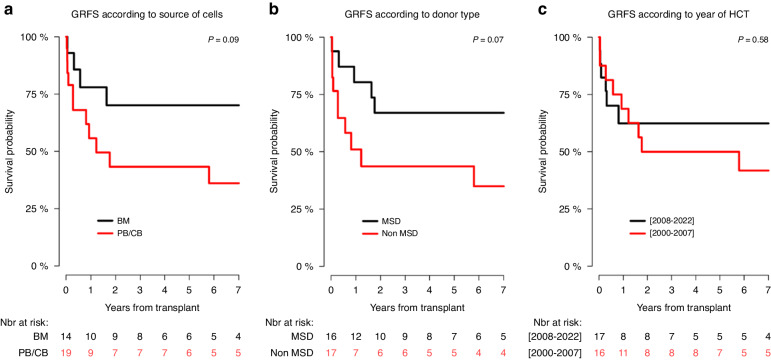
GvHD-free relapse-free survival (GRFS) curves. GRFS according to: the source of haematopoietic cells (**a**), donor of haematopoietic cells (**b**), and transplant period (**c**).

**Fig. 6:**
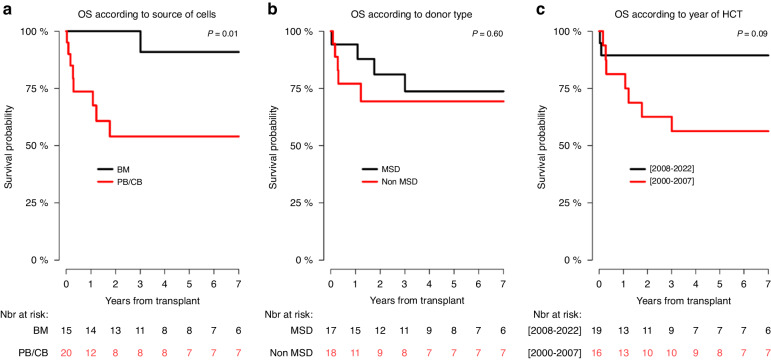
Overall survival (OS) curves. OS according to: the source of haematopoietic cells (**a**), donor of haematopoietic cells (**b**), and transplant period (**c**).

In univariate analysis, the 6-year RI, PFS, GRFS, and OS probability in children transplanted from MSD in comparison with those transplanted from non-MSD were not significantly different (Table 6; Figs. 3, 4, 5, 6), although the 6-year GRFS probability after undergoing MSD-HCT was almost two times higher (67%; 37.9–84.7) than after non-MSD transplantation (34.9%; 12.4–78.1) (*p* = 0.07).

In relation to the source of the haematopoietic cells, the 6-year PFS in children obtaining bone marrow (85.1%; 52.3–96.1) was significantly higher than in those obtaining haematopoietic cells from other sources (50.8%; 26.3–71) (*p* = 0.03) (Table 6; Fig. 4), and the 6-year OS in children receiving bone marrow (90.9%; 50.8–98.7) was significantly higher than in children transplanted with haematopoietic cells from other sources (54%; 28.1–74.2) (*p* = 0.01) (Table 6; Fig. 6). Apart from that, the 6-year GRFS after undergoing a bone marrow transplantation (70.1%; 38.5–87.6) was close to two times higher than that observed for haematopoietic cells transplantation from other sources (36.1%; 14.5–58.4), however, the difference was not significant (*p* = 0.09).

With regard to the time period, the 6-year RI, PFS, GRFS, and the OS in children transplanted between 2008 and 2022 were more favourable than in those transplanted within the period 2000–2007, but the differences were not significant (Table 6; Figs. 4, 5, 6).

## Discussion

Comparing the paediatric population with the population of adults, the *BCR::ABL1*-neg MPNs are not only much rarer, but also in more than 50% of paediatric patients these neoplasms are triple-negative, i.e., without the *JAK2*, *CALR,* or *MPL* mutations, that drive *BCR::ABL1*-neg MPNs in the absolute majority of adult patients [6, 7, 9],

The rarity of *BCR::ABL1*-neg MPNs in children and adolescents severely limits the opportunities to perform clinical studies on these malignancies in paediatric population, and as a consequence, in contrast to the adult population, there are still no established specific diagnostic and prognostic criteria and clear treatment recommendations based on these criteria, including indications for allo-HCT in paediatric patients suffering from *BCR::ABL1*-neg MPN. For these reasons, there have been no prospective or even retrospective studies on the long-term outcomes of allo-HCT in a representative group of paediatric patients with *BCR::ABL1*-neg MPNs, including PMF and post-ET/PV MF.

PMF and post-ET/PV MF are the *BCR::ABL1*-neg MPNs categories with the worst survival rates in adults and can only be cured by allo-HSCT, which can induce molecular remission and resolution of bone marrow fibrosis (10). In the paediatric population PMF and post-ET/PV MF are the rarest category of *BCR::ABL1*-neg MPNs [5–7], and the reports published so far on results of allo-HCT in these patients remain scanty and casuistic [11–14].

The current retrospective study on allo-HCT outcomes in children and adolescents transplanted for PMF or post-ET/PV MF was conducted on behalf of the EBMT Paediatric Diseases Working Party and based on data collected in the EBMT Registry. To date the studied group is the largest cohort of children and adolescents with myelofibrosis (MF) receiving allo-HCT ever collected and analysed.

Previously, the largest group of children with primary myelofibrosis treated and cured with allo-HCT was published by Hussain et al. [13]. This cohort consisted of eight patients under two years of age diagnosed during infancy. However, five of these patients had parents with close consanguinity, and two of them had a strong family history of infant myelofibrosis with recurrent early childhood deaths, which might suggest a congenital predisposition. Apart from this report there have been several other reports of familial cases of infant myelofibrosis primarily in regions of the world with a high level of consanguinity [11, 19]. All the familial cases presented at a very young age and had poor outcomes without HSCT. Thus, the cohort described by Hussein et al. [13] seems to be a very specific one that does not necessarily reflect the biology and clinics of PMF and post-ET/PV MF in the whole paediatric population.

Although, allo-HCT can cure a substantial proportion (55%) of adult patients with PMF or post-ET/PV MF, but is still not universally applicable due to risk of its severe toxic, immunological and infectious complications, which leads to therapy-related morbidity and mortality [10, 20].

Therefore, to determine the prognosis and indications for allo-HCT in adult patients with myelofibrosis, the risk scores taking into account patient- and disease-specific risk factors are currently being developed, which also include the molecular profile [21–24]. In contrast, in children and adolescents with myelofibrosis, the specific prognostic factors have not yet been investigated and in consequence, there are not yet any paediatric risk-scores that can be used to determine the indications for allo-HCT in paediatric patients.

In the studied group of paediatric patients transplanted for myelofibrosis, post-ET/PV MF was a rare indication (5.7%) for allo-HCT, and it was a much less common indication for transplantation than reported in adult patients transplanted for myelofibrosis (21.8%) [20].

The majority of the studied paediatric patients with myelofibrosis were male patients (65.7%) and this may indicate that in the paediatric population, myelofibrosis occurs more often in male patients than in female as opposed to the proportions observed between male and female paediatric patients suffering from essential thrombocythaemia [2, 6, 7, 9]. Indeed, the male predominance (63.2%) among 19 paediatric patients with primary myelofibrosis was also observed by DeLario et al. [25] as well as in adults who underwent allo-HCT for myelofibrosis between 1995 and 2018 (62.8%) [20].

Our study group was characterised by a young median age at diagnosis (3.4 years) and at transplantation (5.2 years). DeLario et al. [25] reported even younger median age (14 months) in 19 pediatric patients with primary myelofibrosis. Such a young age of these patients may indicate the congenital nature of myelofibrosis and the need for molecular studies in these patients to identify specific genetic mutations to guide prognosis and treatment, including allogeneic hematopoietic stem cells;

Apart from the young age of the patients, the median time between diagnosis and transplantation was very short (7.1 months), and much shorter than reported in adults (31.1 months) [20]. The short median time between diagnosis and transplantation may be related to the symptoms of fast progression of the disease, but the median blood counts prior to the start of conditioning regimen for transplantation were only moderately reduced, however, their ranges were wide.

The rate of the Lansky performance status below 90 in the studied children was somewhat lower (25%) than in adults (32.5%), in whom it was identified as one of the factors associated with a worse NRM and OS [20].

Half of the patients from the studied cohort were transplanted from MSD and the second half from non-MSD. The number of patients who underwent allo-HCT between 2000 and 2007 was not significantly different from the number of patients transplanted between 2008 and 2022 (16 vs 19; *p* = 0.73). Thus, there was a unique opportunity to compare allo-HCT outcomes in paediatric patients transplanted for myelofibrosis from MSD and from non-MSD as well as in those transplanted between 2000 and 2007 and between 2008 and 2022.

In the children transplanted from MSD the median age at diagnosis and at transplantation as well as the Lansky score below 90 were not different to those observed in the children transplanted from non-MSD.

Among the differences between these two subpopulations, the median time between diagnosis and transplantation was significantly shorter in the case of MSD-HCT (4.6 months) than in case of non-MSD-HSCT (10.3 months) (*p* = 0.004), and bone marrow was a significantly more frequent source of haematopoietic cells (HCs) for MSD-HCT (64.7%) than for non-MSD-HCT (22.2%) (*p* = 0.006). In the case of adults transplanted for myelofibrosis, the peripheral blood was the predominant source of HCs (in total 88.9%) irrespectively of the donor type [20].

A myeloablative conditioning regimen was given to the vast majority of patients (91.2%). Between 2000 and 2007, it was usually a busulfan-based regimen (81.2%) and no patients obtained a treosulfan-based regimen, whereas between 2008 and 2022, around one third of patients (36.4%) received treosulfan-based regimen, but at this point due to the low number of studied patients it was impossible to compare the transplantation outcomes achieved in patients who received a busulfan-based preparative regimen with those who received a treosulfan-based regimen. However, when looking for the optimal conditioning regimen for paediatric patients with MF, it will be important to perform such a comparison in the future, especially in the context of particulary favourable disease-free survival observed after a treosulfan-based regimen in children with myeloid malignancies along with its reduced organ toxicity, and satisfactory myeloablative and immunosuppressive effects [26].

The issue of the optimal conditioning regimen for adult patients with myelofibrosis remains unclear despite the significant experience in the field, however, Murthy et al. [27] recently demonstrated superior outcomes in a retrospective CIBMTR analysis of 872 patients with conditioning consisted of busulfan and fludarabine in both myeloablative and reduced intensity settings.

While discussing the issue of the optimal conditioning regimen for the children with myelofibrosis an attention can be also drawn to the fact that more than half of the studied children (54.3%) were conditioned for transplantation with busulfan combined with cyclophosphamide, while according to the article by Murthy et al. [27] cited above, in adult patients transplanted for myelofibrosis after myeloablative conditioning based on busulfan and cyclophosphamide the engraftment rates were significantly worse and the risk of acute GvHD grade II-IV significantly higher than observed after myeloablative conditioning based on busulfan and fludarabine. Thus, when looking in the future for the optimal conditioning regimen for paediatric patients with MF, it will be important to evaluate the outcomes of allo-HCT in children achieving the treosulfan-based or busulfan-based conditioning regimen containing fludarabine.

Generally, it is thought that the risk of graft failure and poor graft function in patients transplanted for myelofibrosis is significant due to inflammation, elevated proinflammatory cytokines, fibrosis, and often osteosclerosis within the marrow niche along with splenic sequestration [16, 28]. In the studied cohort of children the day +60 neutrophil and platelet engraftment rates were 85.7% and 78.1%, respectively, and these rates were very similar to those reported by Murthy et al. [27] in adult patients transplanted for myelofibrosis after undergoing myeloablative conditioning based on busulfan and cyclophosphamide (87.2% and 83.7%, respectively), which were significantly worse than the rates observed after myeloablative conditioning based on busulfan and fludarabine (95.2% and 86.1%, respectively). Indeed, as mentioned above, more than half of the studied children (54.3%) were conditioned for transplantation with busulfan and cyclophosphamide without fludarabine. It is also worth noting that Murthy et al. [27] did not observe a difference in the engraftment rates between patients receiving a reduced intensity regimen or myeloablative regimen. In our cohort of patients the non-myeloablative, reduced intensity regimen was used only in three (8.6%) of them. In contrast, as many as 56.5–63.1% of adult recipients with myelofibrosis received the reduced intensity conditioning regimen [20, 27].

In patients undergoing allo-HCT for myelofibrosis, a splenomegaly is also considered to have an impact on engraftment and graft function. Therefore, a splenectomy is an option before transplantation, but a high morbidity and mortality related to splenectomy have been reported, and for this reason, splenectomy is not recommended [10, 29, 30]. In the children studied, a palpable spleen was observed prior to the start of conditioning in almost half them (47.8%), but a splenectomy was performed in only one.

GvHD prophylaxis was almost exclusively CsA-based. T-cell depletion in vivo was used in the absolute majority (88.2%) of patients from the non-MSD-HSCT group, but also in more than half (56.2%) of patients transplanted from MSD, and what more CsA along with short-course methotrexate was administered in the majority of patients who underwent MSD-HCT, while only in around one third of patients who underwent non-MSD-HCT.

The overall day +100 incidence of aGvHD grade II–IV was 39.4%, thus somewhat higher than observed in adults transplanted for myelofibrosis (28–35%) [20], which could be related to the conditioning regimen based on busulfan and cyclophosphamide used in more than half of the studied children, because it was found in adults transplanted for myelofibrosis that the risk of aGvHD grade II–IV and grade III–IV was significantly higher in patients who underwent myeloablative conditioning consisting of busulfan and cyclophosphamide (58.9% and 32.6%, respectively) in comparison with those receiving myeloablative conditioning consisting of busulfan and fludarabine (34.4% and 11.9%, respectively) [27]. In our study group, the day +100 incidence of aGvHD grade II–IV was significantly lower after MSD-HCT (18.8%) than after non-MSD-HCT (58.8%) (*p* = 0.01), and it cannot be ruled out that at least to some extent it could be related to T-cell depletion in vivo and a short-course methotrexate also used in more than half recipients of HCs from MSD.

In the studied cohort of children, the overall 6-year occurrence of cGvHD (16.7%) and extensive cGvHD (14.6%) were several times lower than reported by McLornan et al. [20] in adults transplanted for myelofibrosis. In contrast to the relationship observed by Murthy et al. [27] between the type of conditioning regimen and the risk of severe aGvHD, these authors could not find a significant association between the conditioning regimen used and the incidence of cGvHD in adults.

In the studied group of paediatric patients the incidence of aGvHD III–IV, cGvHD, and extensive cGvHD after undergoing MSD-HCT was lower than observed after undergoing non-MSD-HSCT, but the differences were not significant.

In the analysed cohort, the cumulative incidence of NRM after 6 years was 18%, while McLornan et al. [20] observed a 30% NRM after three years in adults. Graft failure and aGvHD were the exclusive causes of NRM in the studied children, and it speaks also to a need to optimise the conditioning regimen for allo-HCT and GvHD prophylaxis in children with myelofibrosis. In addition, it is worth noting that in the studied paediatric cohort NRM occurred exclusively in children who received HCs from peripheral blood or cord blood. There were no deaths related to infectious complications or conditioning regimen organ toxicity, whereas in reported adults NRM was related to infections and also to GvHD [20].

For the whole study group the 6-year RI was 15.9%, the PFS was 66.1%, the GRFS was 50%, and the OS was 71.1%. For comparison, in the cohort of adults transplanted between 1995 and 2018 for myelofibrosis studied by McLornan et al. [20] the 3-year RI was 21–24%, the RFS was 47–50%, and the OS was 55–60%. In the studied children transplanted with bone marrow, the 6-year PFS and the 6-year OS were significantly higher than in the children who received HCs from other sources, namely 85.1% vs 50.1% (*p* = 0.03) and 90.9% vs 54% (*p* = 0.01), respectively. Thus, taking into the consideration significantly lower NRM along with significantly higher PFS and OS in children transplanted for myelofibrosis with bone marrow, it can be concluded that in paediatric patients with myelofibrosis the bone marrow should be the recommended source of HCs for allo-HCT.

Unlike the case of the studied paediatric patients, McLornan et al. [20] did not identify an impact of the HCs source on the survival outcomes in adults transplanted for myelofibrosis between 1995 and 2018.

In contrast to adult patients transplanted for myelofibrosis between 1995 and 2018 [20], comparing the outcomes in the studied children in relation to the transplant period, the 6-year NRM, RI, PFS, GRFS, and OS in the children transplanted between 2008 and 2022 – despite the trend towards improvement – were not significantly better than those observed in children transplanted between 2000 and 2007 indicating a need to improve the transplant procedure used in children with myelofibrosis.

Several limitations of this study can be recognized, including the retrospective nature of the analysis, small size of the studied cohort of paediatric patients, lack of data on pretransplant treatment, lack of data on mutational status, and lack of comprehensive marrow status data at the time of the allo-HCT.

On the other hand, to date, this is the largest and the first one multicentre study on transplant-specific characteristics and outcomes of allo-HCT for myelofibrosis in paediatric patients. The follow-up time is long and the analysis supports the potentially curative role of allo-HCT for myelofibrosis in children and adolescents. In addition, the study identifies problems related to as extremely rare neoplasm as the myelofibrosis in childhood is, especially in the context of allo-HCT. Namely, there is a lack of comprehensive knowledge about molecular biology of paediatric *BCR::ABL1*-neg MPNs, including myelofibrosis, and therefore, there is a lack of prognostic factors and prognostic-scores, and in consequence there is a lack of clear indications to assure appropriate selection of paediatric patients for allo-HCT.

In conclusion, this first multicenter study on outcomes of allo-HCT in children with myelofibrosis proves feasibility and curative effect of transplantation in these children, suggests that bone marrow transplantation is associated with better outcomes, and indicates the need for further studies to develop the optimal pretransplant, transplant, and posttransplant allo-HSCT procedures as it takes place in adult patients with myelofibrosis [27, 28, 31]. Taking into consideration the extremely rare occurrence of myelofibrosis in the paediatric population, a prospective, randomised clinical trials seem to be unrealistic, but a prospective observational study could be an acceptable, feasible, and effective compromise between a retrospective study and a prospective, randomized clinical trial.

## Data Availability

Data are available on request to the EBMT PDWP.

## References

[1] Arber DA, Orazi A, Hasserjia RP, Borowitz MJ, Calvo KR, Kvasnicka H-M, et al. International Consensus Classification of Myeloid Neoplasms and Acute Leukemias: integrating morphologic, clinical, and genomic data. Blood. 2022;140:1200–28. 35767897 10.1182/blood.2022015850 PMC9479031

[2] Hofmann I. Myeloproliferative neoplasms in children. J Hematopathol. 2015;8:143–57. 10.1007/s12308-015-0256-1 PMC4655194 26609329

[3] Grinfeld J, Nangalia J, Green AR. Molecular determinants of pathogenesis and clinical phenotype in myeloproliferative neoplasms. Haematologica. 2017;102:7–17. 27909216 10.3324/haematol.2014.113845 PMC5210228

[4] Vannucchi AM, Gugielmelli P Molecular prognostication in Ph-negative MPNs in 2022. ASH Education Program - Hematology 2022; pp. 225-34. 10.1182/hematology.2022000339 PMC9820187 36485130

[5] Tefferi A, Barbui T. Polycythemia vera and essential thrombocythemia: 2021 update on diagnosis, risk stratification and management. Am J Hematol. 2020;95:1599–613. 32974939 10.1002/ajh.26008

[6] Ianotto J-C, Curio-Garcia N, Lauermanova M, Radia D, Kiladjian J-J, Harrison CN. Characteristics and outcomes of patients with essential throbocythemia or polycythemia vera diagnosed before 20 years of age: a systemic review. Haematologica. 2019;104:11580–1588. 10.3324/haematol.2018.200832 PMC6669170 30679326

[7] Sobas M, Kiladijan JJ, Beauverd Y, Curto-Garcia N, Sadjadian P, Shih LY, et al. Real-world study of children and young adults with myeloproliferative neoplasms: identifying risks and unmet needs. Blood Adv. 2022;6:5171–83. 35802458 10.1182/bloodadvances.2022007201 PMC9631631

[8] Kucine N, Al.-Kawaaz M, Hajje D, Bussel J, Orazi A. Difficulty distinguishing essential thrombocythaemia from polycythaemia vera in children with *JAK2* V617F-positive myeloproliferative neoplasms. Br J Haematol. 2019;185:136–42. 29767848 10.1111/bjh.15386 PMC6239998

[9] Kucine N. Myeloproliferative neoplasms in children, adolescents and young adults. Curr Hematol Malig. 2020;15:141–6. 10.1007/s11899-020-00571-8 PMC7234912 32172359

[10] Kröger N, Chalandon Y Myeloproliferative Neoplasms. In: Varreras E, Dufour C, Mohty M, Kröger N (Eds.). EBMT Handbook – Hematopoietic Stem Cell Transplantation and Cellular Therapies. Springer Open, 2019, pp. 569-78. 32091673

[11] Domm J, Calder C, Manes B, Crossno C, Correa H, Frangoul H. Unrelated stem cell transplant for infantile idiopathic myelofibrosis. Pediatr Blood Cancer. 2009;52:893–5. 19241452 10.1002/pbc.21910

[12] Shaikh F, Naithani R, Kirby-Allen M, Doyle L. Allogeneic cord hematopoietic stem cell transplantation in an infant with primary myelofibrosis. J Pediatr Hematol Oncol. 2012;34:199–201. 10.1097/MPH.0b013e3182346cc5 22246154

[13] Hussein AA, Domm HT, Al-Zaben A, Frangoul H. Allogeneic hematopoietic stem cell transplantation for infants with idiopathic myelofibrosis. Pediatr Transplantation. 2013;17:815–9. 10.1111/petr.12148 24102929

[14] Mitton B, de Oliveira S, Pullarkat ST, Moore TB. Stem cell transplantation in primary myelofibrosis of childhood. J Pediatr Hematol Oncol. 2013;35:e120–e122. 23511496 10.1097/MPH.0b013e31828800cc

[15] Valcárcel D, Sureda A Graft failure. In: Carreas E, Dufour C, Mohty M, Kröger N (eds). The EBMT Handbook. Springer International Publishing, 2019. Pp 307-13. http://link.springer.com/10.1007/978-3-030-02278-5_41.

[16] McLornan DP, Boluda JCH, Czerw T, Cross N, Deeg HJ, Ditschkowski M, et al. Allogeneic haematopoietic cell transplantation for myelofibrosis: proposed definitions and management strategies for graft failure, poor graft function and relapse: best practice recommendations of the EBMT Chronic Malignancies Working Party. Leukemia. 2021;35:2445–59. 34040148 10.1038/s41375-021-01294-2

[17] Przepiorka D, Weisdorf D, Martin P, Klingemann HG, Beatty P, Hows J, Thomas ED. Consensus conference on acute GVHD grading. Bone Marrow Transplant. 1995;15:825–8. 7581076

[18] Filipovich AH, Weisdorf D, Pavletic S, Socie G, Wingard JR, Lee SJ, et al. National Institutes of Health consensus development project on criteria for clinical trials in chronic graft-versus-host disease: I. Diagnosis and staging working group report. Biol Blood Marrow Transplant. 2005;11:945–56. 16338616 10.1016/j.bbmt.2005.09.004

[19] Rosbach HC. Familial infantile myelofibrosis as an autosomal recesseive disorder: preponderance among children from Saudi Arabia. Pediatr Hematol Oncol. 2006;23:453–4. 16728367 10.1080/08880010600623240

[20] McLornan D, Eikema DJ, Czerw T, Kröger N, Koster R, Reinhardt HC, et al. Trends in allogeneic haematopoietic cell transplantation for myelofibrosis in Europe between 1995 and 2018: a CMWP of EBMT retrospective analysis. Bone Marrow Transplant. 2021;56:2160–72. 33911203 10.1038/s41409-021-01305-x

[21] Guglielmelli P, Lasho TL, Rotunno G, Mudireddy M, Mannarelli C, Nicolosi M, et al. MIPSS70: Mutation-enhanced International Prognostic Score System for transplantation-age patients with primary myelofibrosis. J Clin Oncol. 2018;36:310–8. 29226763 10.1200/JCO.2017.76.4886

[22] Kröger NM, Deeg JH, Olavarria E, Niederwieser D, Bacigalupo A, Barbui T, et al. Indication and management of allogeneic stem cell transplantation in primary myelofibrosis: a consensus process by an EBMT/ELN international working group. Leukemia. 2015;29:2126–33. 26293647 10.1038/leu.2015.233

[23] Passamonti F, Giorgino T, Mora B, Guglielmelli P, Rumi E, Maffioli M, et al. A clinical-molecular prognostic model to predict survival in patients with post polycythemia vera and post essential thrombocythemia myelofibrosis. Leukemia. 2017;31:2726–31. 28561069 10.1038/leu.2017.169

[24] Gagelmann N, Ditschkowski M, Bogdanov R, Bredin S, Robin M, Cassimat B, et al. Comprehensive clinical-molecular transplant scoring system for myelofibrosis undergoing stem cell transplantation. Blood. 2019;133:2233–342. 30760453 10.1182/blood-2018-12-890889

[25] DeLario MR, Sheehan AM, Ataya R, Bertuch AA, Vega CII, Webb CR, et al. Clinical, histopathologic, and genetic features of pediatric primary myelofibrosis – an entity different from adults. Am J Hematol. 2012;87:461–5. 22389089 10.1002/ajh.23140

[26] Wachowiak J, Sykora K-W, Cornish J, Chybicka A, Kowalczyk JR, Gorczyńska E, et al. Treosulfan-based preparative regimens for allo-HSCT in childhood hematological malignancies: a retrospective study on behalf of the EBMT Pediatric Diseases Working Party. Bone Marrow Transplant. 2011;4:1510–8. 10.1038/bmt.2010.343 21297673

[27] Murthy GSG, Kim S, Estrada-Merly N, Abid MB, Aljurf M, Assal A, et al. Association between the choice of the conditioning regimen and outcomes of allogeneic hematopoietic cell transplantation for myelofibrosis. Haematologica. 2023;108:1900–8. 36779595 10.3324/haematol.2022.281958 PMC10316233

[28] Perram J, Ross DM, McLornan D, Gowin K, Kröger N, Gupta V, et al. Innovative strategies to improve hematopoietic stem cell transplant outcomes in myelofibrosis. Am J Hematol. 2022;97:1464–77. 35802782 10.1002/ajh.26654 PMC9796730

[29] Tefferi A, Mesa RA, Nagorney DM, Schroeder G, Silverstein MN. Splenectomy in myelofibrosis with myeloid metaplasia: a single-institution experience with 223 patients. Blood. 2000;95:2226–33. 10733489 10.1182/blood.V95.7.2226

[30] Malato A, Rossi E, Tiribelli M, Mendicino F, Pugliese N. Splenectomy in myelofibrosis: indications, efficacy, and complications. Clin Lymphoma Myeloma Leuk. 2020;20:588–95. 32482540 10.1016/j.clml.2020.04.015

[31] Gagelmann N, Kröger N. Improving allogeneic stem cell transplantation in myelofibrosis. Int J Hematol. 2022;115:619–25. 35419771 10.1007/s12185-022-03340-w

